# Cardiac magnetic resonance in heart transplant recipients: histological, clinical and cell-free DNA validation

**DOI:** 10.1093/ehjci/jeaf145

**Published:** 2025-05-19

**Authors:** Marko A Taipale, Markku O Pentikäinen, Laura A Martelius, Aino Mutka, Soili I Kytölä, Matti Kankainen, Juha I Peltonen, Simo O Syrjälä, Arttu J I Lahtiharju, Jyri J Lommi, Timo J Jahnukainen, Karl B Lemström, Tiina H Ojala

**Affiliations:** Department of Pediatric Cardiology, Pediatric Research Center, New Children’s Hospital, University of Helsinki and Helsinki University Hospital, PO Box 281, Stenbäckinkatu 11, Helsinki FI-00029 HUS, Finland; Department of Cardiology, Heart and Lung Center, Helsinki University Hospital and University of Helsinki, Helsinki, Finland; Department of Radiology, Helsinki University Hospital and University of Helsinki, Helsinki, Finland; Department of Pathology, Helsinki University Hospital and University of Helsinki, Helsinki, Finland; Laboratory of Genetics, HUS Diagnostic Center, Helsinki University Hospital and University of Helsinki, Helsinki, Finland; Laboratory of Genetics, HUS Diagnostic Center, Helsinki and Uusimaa Hospital District, Hematology Research Unit Helsinki, Translational Immunology Research Program, University of Helsinki, Helsinki, Finland; Department of Radiology, Helsinki University Hospital and University of Helsinki, Helsinki, Finland; Department of Cardiothoracic Surgery, Heart and Lung Center, Helsinki University Hospital and University of Helsinki, Helsinki, Finland; Department of Cardiothoracic Surgery, Heart and Lung Center, Helsinki University Hospital and University of Helsinki, Helsinki, Finland; Department of Cardiology, Heart and Lung Center, Helsinki University Hospital and University of Helsinki, Helsinki, Finland; Department of Pediatric Nephrology and Transplantation, Helsinki University Hospital and University of Helsinki, Helsinki, Finland; Department of Cardiothoracic Surgery, Heart and Lung Center, Helsinki University Hospital and University of Helsinki, Helsinki, Finland; Department of Pediatric Cardiology, Pediatric Research Center, New Children’s Hospital, University of Helsinki and Helsinki University Hospital, PO Box 281, Stenbäckinkatu 11, Helsinki FI-00029 HUS, Finland

**Keywords:** CMR, EMB, T1 mapping, T2 mapping, heart transplantation, rejection

## Abstract

**Aims:**

Cardiac magnetic resonance (CMR) presents a promising non-invasive method for evaluating acute rejection in heart transplant recipients. As indicators of myocardial injury, T1 and T2 mapping values are crucial for comprehending rejection patterns in heart transplants. This study aims to define CMR T1 and T2 mapping values in heart transplant patients both with and without acute rejection.

**Methods and results:**

In this blinded prospective study, we analysed CMR data from 244 scans of 58 paediatric and adult heart transplant recipients, 1–24 months post-transplant. Rejection status was defined by endomyocardial biopsy, clinical data, and donor-derived cell-free DNA (dd-cfDNA). Over the 24 months post-transplant, global T1 and T2 values decreased significantly (T1: *β* = −8.9/log(month), *P* < 0.001; T2: *β* = −0.5/log(month), *P* < 0.001) demonstrating the gradual recovery from transplant-related myocardial injury. During acute rejection, T1 values significantly increased compared to rejection-free studies in both children [estimates at 1 month post-transplant 1188 ms (95% CI: 1161–1215) vs. 1079 ms (95% CI: 1061–1097), *P* < 0.001] and adults [1087 ms (95% CI: 1045–1129) vs. 1016 ms (95% CI: 1005–1027), *P* = 0.007]. T1 and T2 values were positively associated with dd-cfDNA (*P* < 0.001 and *P* = 0.014, respectively), and T2 values with worse left ventricular global longitudinal strain (*P* < 0.001).

**Conclusion:**

We provide essential T1 and T2 mapping values across cardiac segments, as well as left ventricular myocardial strain, both with and without acute rejection. These findings establish a reliable foundation for non-invasive heart transplant rejection screening.

**Clinical trial registration:**

ClinicalTrials.gov Identifier: NCT04311346

## Graphical Abstract

**Graphical Abstract:**
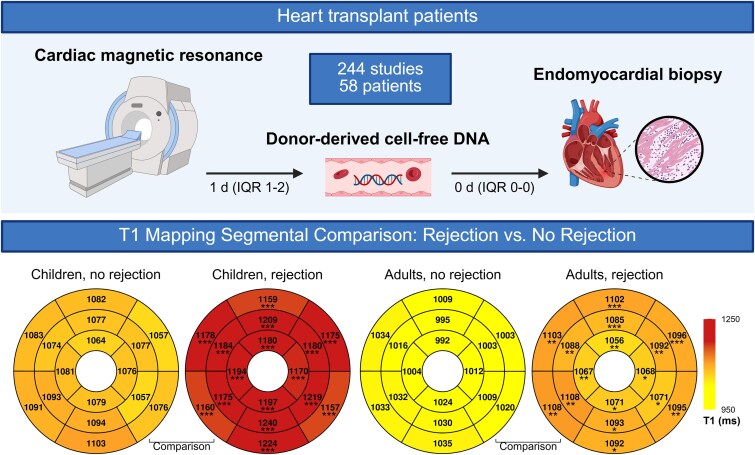
T1 mapping in cardiovascular magnetic resonance in heart transplants. Top panel: flowchart of the study. Bottom panel: linear mixed-effects model estimates of T1 values and segmental comparisons between rejection and rejection-free groups in children and adults at 1 month post-transplant. *P < 0.050, **P < 0.010, ***P < 0.001.

## Introduction

The field of heart transplantation has long faced the challenge of accurately and promptly diagnosing organ rejection. Traditionally, endomyocardial biopsy (EMB) has been the gold standard for rejection surveillance,^1^ despite its invasive nature and limitations in reliability.^2^

Cardiac magnetic resonance imaging (CMR) with left ventricular parametric mapping, focusing on T1 and T2 times,^3–9^ as well as strain assessment^10,11^ has emerged as a non-invasive alternative to traditional rejection screening methods. Within CMR, an increase in myocardial T1 and T2 values has been associated with oedema and inflammation, which are histological changes in heart transplant rejection.

While some studies suggest that CMR may replace biopsies for monitoring rejection, caution persists regarding the interpretation of baseline parameters in CMR following heart transplantation. The heart is a complex organ typically divided into 16 segments, and rejection may not be uniformly distributed across these areas. However, previous CMR studies have primarily focused on septal segments for myocardial analysis, while only EMBs have been used to confirm the rejection-free status. Thus, there is a significant gap in knowledge, as studies incorporating the entire left ventricle for CMR analysis and utilizing all contemporary rejection markers for a comprehensive assessment of rejection are notably lacking.

In this blinded prospective study, we aimed to characterize myocardial T1/T2 mapping and strain, common CMR markers of rejection, in paediatric and adult heart transplant patients. We present T1/T2 values over 24 months post-transplant, demonstrate differences between groups with acute rejection and no rejection, as well as between adults and children. Additionally, we present T1/T2 values globally and across 16 cardiac segments, and explore associations with underlying factors. Rejection status was defined using a combination of histological analysis, clinical findings, cardiac function assessments, and donor-derived cell-free DNA (dd-cfDNA), a clinically validated non-invasive marker of graft injury.^12,13^ We hypothesized that myocardial T1 and T2 values are elevated during acute rejection and associate positively with dd-cfDNA levels, reflecting ongoing graft injury.

## Methods

### Study design

The study was conducted at two tertiary medical centres (Heart and Lung Center and New Children’s Hospital at Helsinki University Hospital), which are responsible for all adult and paediatric heart transplantations in Finland. Children under 18 were scanned with Vendor 1, and adults aged 20 or older with Vendor 2. Youths in the transition phase, aged 18–20, were classified as either children or adults based on the vendor used for their scan. Among heart transplant patients between March 2020 and December 2023, 41 of 59 adults (69%) and 17 of 27 children (63%) participated in this prospective study (NCT04311346). Patients were enrolled shortly after transplantation by the transplant co-ordinator. The study was approved by the Ethics Committee of the Hospital District of Helsinki, and Uusimaa (HUS) (HUS/3341/2019) and all participating patients provided written informed consent.

**Figure 1:**
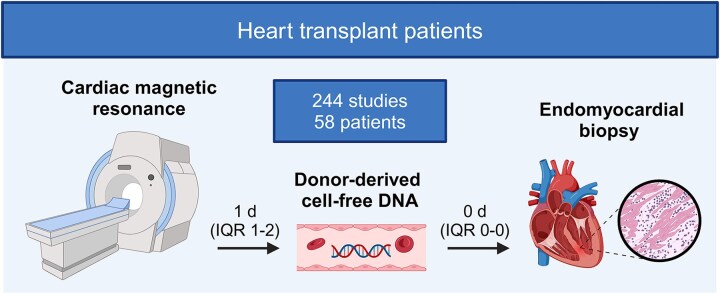
Flowchart of the study.

**Figure 2:**
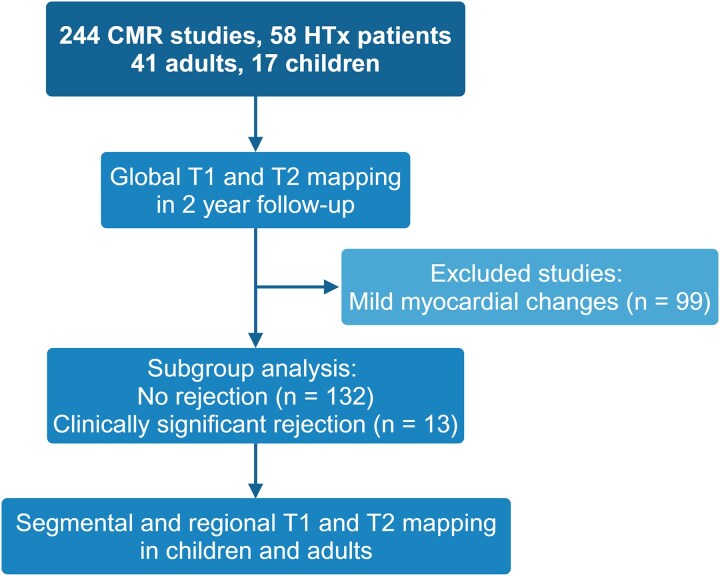
Presentation of CMR studies included in the analyses of heart transplantation patients.

Post-transplant surveillance, including CMR, EMB, and dd-cfDNA testing, was scheduled at 1, 2, 3, 4, 5, 6, and 12 months, and with CMR alone at 24-month point (*Figure 1*). dd-cfDNA testing was not systematically performed in children. Additionally, data from five acute rejections in four children, 3–14 years post-transplant, were included in the analysis. Patients were divided into subgroups at each time point based on the presence or absence of acute rejection (*Figure 2*). Acute rejection was defined based on three parameters: EMB, clinical status, and dd-cfDNA. The three parameters were considered abnormal if: (i) EMB revealed grade ≥2R acute cellular rejection (ACR) or grade ≥1 antibody-mediated rejection (AMR); (ii) symptoms or ultrasound findings indicated clinical rejection; or (iii) dd-cfDNA level was >0.20%. Rejection was defined by the presence of 2 out of 3, or 1 out of 2, abnormal parameters. dd-cfDNA samples were excluded if levels exceeded 0.20 within the first 6 weeks or if cytomegalovirus nucleic acid exceeded 1000 IU/mL, which indicated clinically significant cytomegaloviremia. Inclusion criteria for the subgroup of rejection-free heart transplantation subjects comprised an EMB demonstrating no rejection (ACR grade 0R and pAMR grade 0), no signs of clinical rejection, dd-cfDNA < 0.15%, left ventricular ejection fraction (LVEF) ≥ 40%, and absence of acute rejection within the previous month. CMR scans from patients with rejection in the past month but no ongoing rejection were excluded from the rejection analysis, as T1 and T2 values can remain elevated following a rejection episode. One CMR scan was excluded because of the poor imaging quality. All transplants were from donors after brain death, with no marginal donors identified, and coronary angiography, if conducted, showed normal results.

### CMR acquisition

All CMR studies were acquired with 1.5T magnetic resonance imaging (MRI) scanner [children: Ingenia 1.5T, Philips Healthcare, Best, the Netherlands (Vendor 1); adults: AvantoFit, Siemens Healthineers, Erlangen, Germany (Vendor 2)]. All sequences were scanned with electrocardiogram gating and breath-hold as primary method to suppress motion. The protocols for cine imaging and late gadolinium enhancement (LGE) imaging are described in the Supplementary data online, Supplementary Data.

T1 mapping was acquired in three short-axis planes (base, mid, and apical) by using modified Look-Locker inversion recovery (MOLLI) sequence in systolic and diastolic heart phase. The slice thickness was 6 or 8 mm and a typical acquisition pixel size 2.0 × 2.0 mm or 1.2 × 1.2 mm with paediatric and adult patients, respectively. The imaging was performed without contrast agent.

T2 mapping was performed in identical imaging planes with T1 mapping in systolic and diastolic phases. Paediatric imaging used a GraSE-based sequence with nine echoes (8.8–79.2 ms), 6 mm slice thickness, and 2.2 × 2.2 mm pixel size. Adults underwent a T2-prepared sequence with three echo times (0, 24, and 55 ms) and balanced steady-state free precession readout, with 8 mm slice thickness and 1.9 × 1.9 mm pixel size. A vendor-provided motion correction algorithm was used in adults to reduce motion artefacts.

T1 and T2 relaxation measurement bias between scanners was controlled using an in-house produced gel phantom with inserts of varying T1 and T2 relaxation times, adjusted with ammonium 2-sulphate ((NH4)2Fe(SO4)2) concentration.^14^ Phantom images were acquired using the same setup as for human subjects, with a simulated heart rate of 60/min. The correction factors for T1 and T2, calculated from the average relative difference across inserts, were 1.022 and 1.014, respectively, with Vendor 1 showing higher values before applying the correction factors. Additionally, 11 healthy controls were scanned by both vendors, with correction factors of 1.029 for T1 and 1.029 for T2, which fall within the error margins of phantom measurements. The phantom-derived correction factors were applied in comparisons between children and adults.

### CMR image analyses

From multiple T1 and T2 mapping images, the highest-quality ones were chosen for analysis. Myocardium was delineated manually in basal, mid-ventricular, and apical short-axis slices to calculate T1- and T2-relaxation times of 16 segments according to the left ventricular segmentation model by American Heart Association.^15^ Epi- and endocardial borders were followed carefully to avoid intravoxel contamination at tissue border (see Supplementary data online, *Figure S1*). Blinded to EMB and dd-cfDNA results, one senior cardiologist (T.H.O.; fully certified in ESC CMR CHD, Level 3), interpreted the images. For a more comprehensive evaluation, we categorized segments based on their anatomical locations into anterior, inferior, septal, lateral, basal, mid-ventricular, and apical groups. The extent of LGE was assessed using the full width at half maximum method, with manual adjustments as required (see Supplementary data online, *Figure S2*). Detailed CMR image analysis, including strain measurements, is provided in the Supplementary data online, Supplementary Data.

### EMB, histology and dd-cfDNA

The EMB protocol, as well as the histological and dd-cfDNA methods, is described in detail in the Supplementary data online, Supplementary Data. Briefly, an experienced pathologist, blinded to the CMR and dd-cfDNA results, interpreted EMB samples according to the International Society of Heart and Lung Transplantation (ISHLT) 2005 and 2013 criteria.^16,17^ Venous blood samples were collected for ddPCR-based dd-cfDNA analysis, and dd-cfDNA percentages were calculated by comparing droplets with donor alleles to those with both donor and reference alleles.

### Statistical analysis

Categorical variables are expressed as counts and percentages, while continuous variables are described using medians and inter-quartile ranges (IQRs). T1, T2, and strain values were analysed using linear mixed-effects models with logarithmic time as a covariate in each model. We accounted for repeated measurements with a random intercept for each patient, as well as for measurements from different segments within the same patient in pooled models. Segmental and regional differences in T1 and T2 values were modelled with rejection status as a random slope; however, this was not applied to children in the segmental analysis due to the small sample size. *P*-values for model estimates were determined using the Satterthwaite estimator for degrees of freedom. To improve model fit, T1 and T2 values were winsorized to the 1st and 99th percentiles. To assess both intra- and inter-observer variability of T1 and T2 values, as well as inter-observer variability of LGE, we applied a two-way mixed model with absolute agreement and average measures to calculate the intra-class correlation coefficients (ICC) (3,k). All analyses were conducted with R (R Core Team, Vienna, Austria, 2024) version 4.4.2 using lme4, and lmerTest.

T1 and T2 mapping demonstrated good intra-observer agreement [T1 ICC was 0.81 (95% CI: 0.74–0.86), and T2 ICC was 0.81 (95% CI: 0.73–0.86)] and inter-observer agreement [T1 ICC was 0.71 (95% CI: 0.60–0.79), and T2 ICC was 0.80 (95% CI: 0.73–0.85)] in real life situation. Inter-observer agreement for LGE was good [ICC 0.75 (95% CI: 0.59–0.84)].

## Results

### Patients

In this blinded prospective study (Trial NCT04311346), we analysed CMR data from 244 scans of 58 heart transplant recipients (200 scans from 41 adults and 44 scans from 17 children) in Finland (*Figure 2*). Thirteen acute rejections appeared, five rejections in four children and eight rejections in five adults. At each time point, CMR was first conducted and immediately reported blindly to the electronic medical record (*Figure 1*). Subsequently, dd-cfDNA testing was performed prior to the EMB, which occurred at a median of 1 day (IQR 1–2 days) after the CMR. CMR scan availability per time point ranged from 24 scans at 4 months (41% of patients) to 40 scans at 6 months (69% of patients). Patient characteristics are summarized in *Tables 1* and *2*.

**Table 1:** Baseline patient characteristics

	Paediatric transplant recipients (*n* = 17)	Adult transplant recipients (*n* = 41)	*P*-value
Age at HTx (years)	12.3 (10.3–13.3)	54.7 (45.2–61.7)	**<0**.**001**
Age range (years)	1.8–15.9	17.8–69.2	
Sex, male	7 (41%)	33 (80%)	**0**.**008**
BSA at HTx (m^2^)	1.23 (1.14–1.35)	1.95 (1.81–2.09)	**<0**.**001**
HTx indication			**0**.**044**
DCM	4 (24%)	17 (41%)	
IHD	0	8 (20%)	
HCM	3 (18%)	4 (10%)	
Other CM	4 (24%)	4 (10%)	
CHD	5 (29%)	3 (7%)	
Other	1 (6%)	5 (12%)	
DSA at HTx	6 (40%)	10 (24%)	0.417
VAD before HTx	2 (12%)	15 (37%)	0.116
Donor age (ears)	19 (14–23)	38 (31–46)	**<0**.**001**
Donor cause of death			0.541
Stroke	4 (27%)	19 (46%)	
Head trauma	6 (40%)	13 (32%)	
Anoxia	4 (27%)	6 (15%)	
Other	1 (7%)	3 (7%)	
Ischaemic time (min)	205 (146–251)	186 (117–214)	0.200
Post-HTx ECMO	7 (41%)	5 (12%)	**0**.**034**
Hospital stay (days)	35 (30–54)	38 (20–47)	0.338
No. of scans per patient	2 (1–3)	5 (3–7)	**0**.**002**
EMB, days after CMR	1 (0–2)	1 (1–2)	0.533

Values are median (IQR), or *n* (%). *P*-values were calculated using Mann–Whitney *U* tests for continuous variables and χ^2^ tests for categorical variables. Donor cause of death data is missing for two paediatric patients; the comparison includes 15 children. The bold values denote statistically significant differences.

BSA, body surface area; CHD, congenital heart disease; CM, cardiomyopathy; CMR, cardiac magnetic resonance imaging; DCM, dilated cardiomyopathy; DSA, donor-specific antibody; HCM, hypertrophic cardiomyopathy; HTx heart transplantation; ECMO, extracorporeal membrane oxygenation; EMB, endomyocardial biopsy; IHD, ischaemic heart disease; VAD, ventricular assist device

**Table 2:** Rejection parameters

	Frequency of events
EMB, *n* = 210	
ACR grade 0R	132 (63%)
ACR grade 1R	67 (32%)
ACR grade 2–3R	11 (5%)
pAMR 0	205 (98%)
pAMR 1–3	5 (2%)
Clinical assessment, *n* = 244	
No rejection	233 (95%)
Rejection	11 (5%)
dd-cfDNA, *n* = 164	
<0.15%	144 (88%)
0.15–0.20%	7 (4%)
0.20%	13 (8%)

Overview of rejection parameter distribution.

ACR, acute cellular rejection; AMR, antibody-mediated rejection; dd-cfDNA, donor-derived cell-free DNA; EMB, endomyocardial biopsy.

### T1 maps

**Figure 3:**
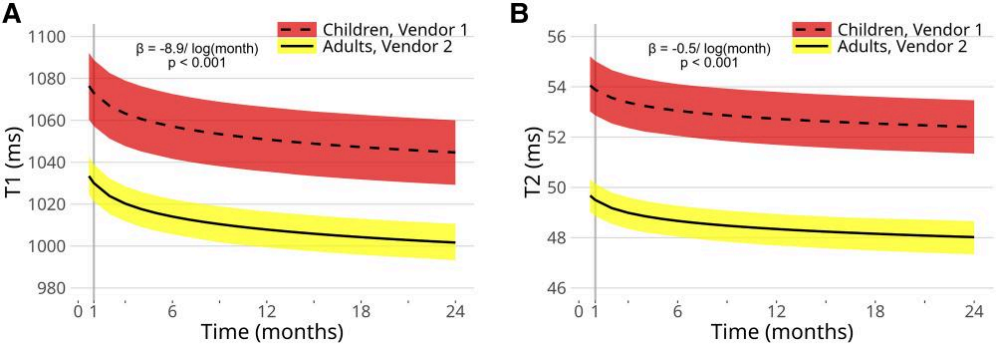
Effect of time on T1 values (*A*) and T2 values (*B*) in a linear mixed-effects (LME) model with logarithmic time for children and adults. Coloured zone illustrates 95% CI.

**Figure 4:**
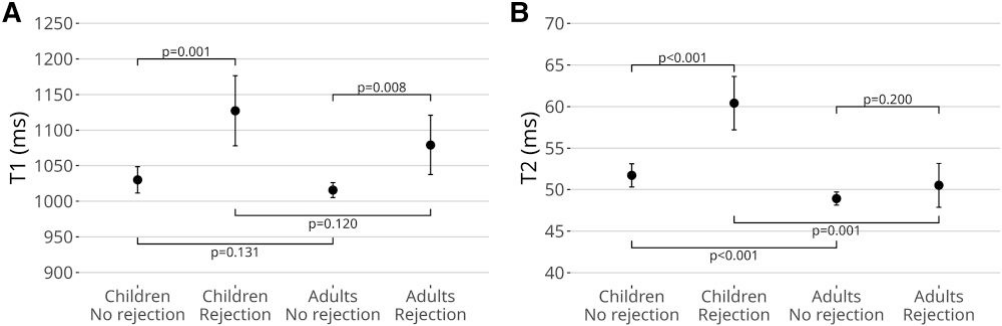
Global T1 and T2 values (95% CI) in rejection vs. non-rejection. LME model estimates and comparisons of vendor-adjusted T1 values (*A*) and T2 values (*B*) in children and adults at 1 month post-transplant. The image shows gender-adjusted values for males.

**Figure 5:**
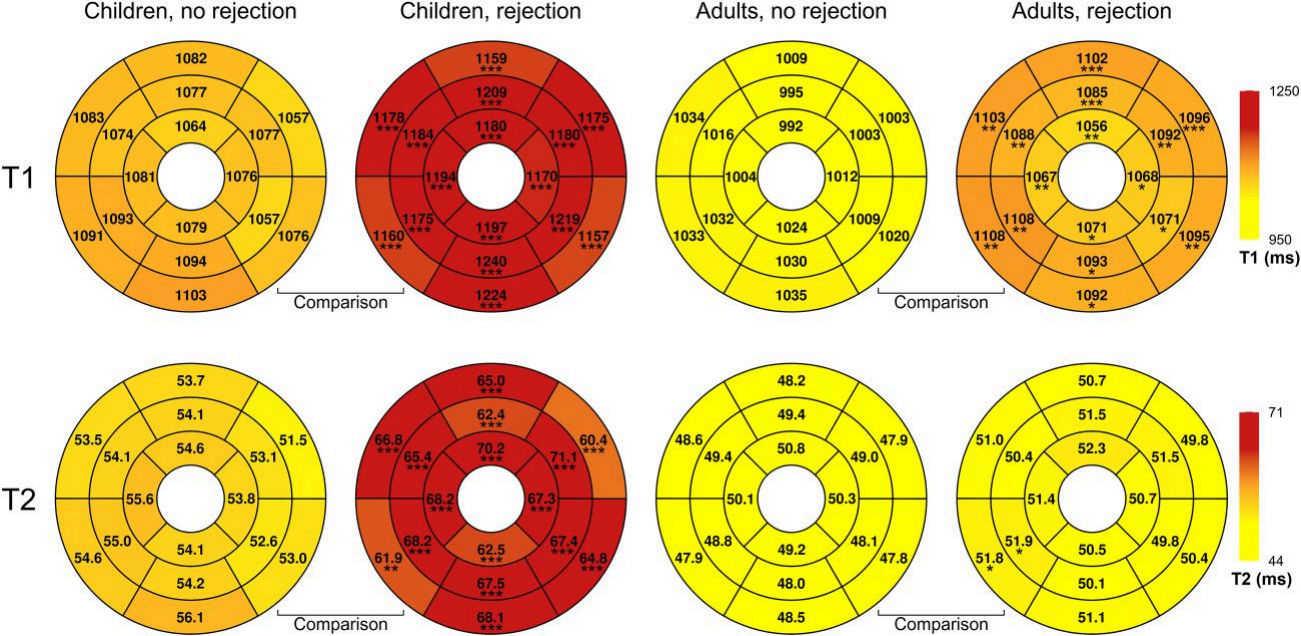
LME model estimates of T1 and T2 values and segmental comparisons between rejection and rejection-free groups in children and adults at 1 month post-transplant. *P < 0.050, **P < 0.010, ***P < 0.001.

**Figure 6:**
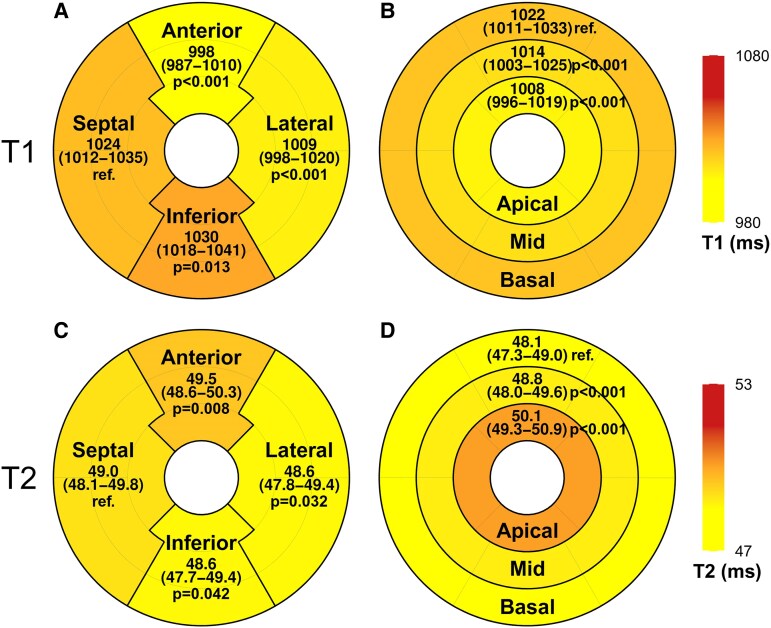
Regional T1 mapping (*A* and *B*) and T2 mapping (*C* and *D*) LME model estimates (95% CI) and comparisons in rejection-free adults at 1 month post-transplant. (*A* and *C*) Septal segments compared to anterior, lateral, and inferior segments. (*B* and *D*) Basal segments compared to mid-ventricular and apical segments.

Over the 24-month study period, T1 values decreased significantly in both children and adults [*β* = −8.9/log(month) (95% CI: −10.4 to −7.5), *P* < 0.001]. (*Figure 3*). After adjusting for post-transplant time, T1 values were higher during acute rejection compared to the rejection-free group in both age groups (*Table 3*, *Figure 4*). Within the rejection group, T1 values were elevated in all left ventricular segments (*Figure 5*). After adjusting for gender, no difference was observed between children and adults in the vendor-corrected values (*Figure 4*). Furthermore, in the rejection-free group, we observed significant variations in T1 values across left ventricular regions, with higher values in the septal and inferior segments in both age groups and basally in adults (*Figure 6* and Supplementary data online, *Figure S3*).

**Table 3:** T1, T2, GLS, and GCS estimates

	No rejection (132 scans)	Rejection (13 scans)	*P*-value
Children	30 scans	5 scans	
T1 (ms)	1079 (1061–1097)	1188 (1161–1215)	**<0**.**001**
T2 (ms)	54.0 (52.2–55.8)	66.1 (63.3–68.9)	**<0**.**001**
GLS (%)	−23.6 (−26.3 to −20.8)	−19.4 (−24.8 to −14.0)	0.159
GCS (%)	−35.8 (−40.2 to −31.4)	−28.3 (−36.9 to −19.7)	0.115
Adults	102 scans	8 scans	
T1 (ms)	1016 (1005–1027)	1087 (1045–1129)	**0**.**007**
T2 (ms)	48.9 (48.1–50.0)	51.0 (48.1–53.8)	0.117
GLS (%)	−22.7 (−24.7 to −20.8)	−19.0 (−23.7 to −14.3)	0.123
GCS (%)	−29.9 (−32.1 to −27.6)	−26.7 (−31.7 to −21.8)	0.210

LME model estimates (95% CI) for no rejection and rejection groups, with *P*-values for group comparisons. T1 and T2 values are from the models at 1 month post-transplant. The bold values denote statistically significant differences.

GCS, global circumferential strain; GLS, global longitudinal strain.

### T2 maps

T2 values exhibited significant decrease during the 24-month surveillance period both in children and adults [*β* = −0.5/log(month) (95% CI: −0.6 to −0.3), *P* < 0.001, *Figure 3*]. After adjusting for time since transplantation, T2 values were higher during acute rejection compared to the rejection-free group in children but not in adults (*Table 3*). In vendor-corrected values, T2 levels were higher in children than in adults (*Figure 4*). In the rejection-free group, significant variations in T2 values were observed across distinct left ventricular regions, with higher values in the septal segments in both age groups and apically in adults (*Figure 6* and Supplementary data online, *Figure S3*).

### Strain

Over the 24-month study period left ventricular global longitudinal strain (GLS) and global circumferential strain (GCS) exhibited no change over time [GLS: *β* = −0.3/log(month) (95% CI: −0.9 to 0.4), *P* = 0.383; GCS: *β* = −0.2/log(month) (95% CI: −1.0 to 0.6), *P* = 0.575]. GLS or GCS showed no significant differences between the rejection group and the rejection-free group in either adults or children (*Table 3*).

### Late gadolinium enhancement

LGE was observed in 26 of the 42 patients (62%) in the first CMR studies with contrast imaging, median 6.4 months (IQR 2.9–12.1 months) after transplantation. The extent of LGE was a median of 6.5%, with 1–4% in 8 patients (31%), 5–9% in 14 patients (54%), and ≥10% in 4 (15%) patients. The median involvement of LGE was significantly higher in the rejection group [8% (IQR 5–12%)] compared to the rejection-free group [2% (IQR 0–7%); *P* < 0.001]. LGE was more often observed in a diffuse/patchy pattern (90%) than in a regional transmural infarct (10%). In the univariate analysis, T1 values were positively associated with the presence of ≥5% LGE [*β* = 16.6 (95% CI: 9.0–24.3), *P* < 0.001].

### Multivariable analysis

In multivariable analysis, vendor-corrected T1 values were positively associated with dd-cfDNA [*β* = 5.24 per 0.1 increase (95% CI: 3.79–6.68), *P* < 0.001] and negatively associated with time since transplantation [*β* = −3.12 per log(month) (95% CI: −5.46 to −0.79), *P* = 0.009] and right ventricular ejection fraction (RVEF) (*β* = −0.60 [95% CI: −1.03 to −0.16], *P* = 0.007) (see Supplementary data online, *Tables S1* and *S2*). Regarding T2 mapping, vendor-corrected values were positively associated with dd-cfDNA [*β* = 0.14 per 0.1 increase (95% CI: 0.03–0.25), *P* = 0.014], and worse GLS [*β* = 0.10 (95% CI: 0.07–0.14), *P* < 0.001]. Furthermore, T2 values were positively associated with donor age (*P* = 0.046), donor sex (female) (*P* = 0.007), and transplantation perfusion time (*P* = 0.013). These associations remained significant after adjusting for dd-cfDNA, GLS, GCS, LVEF, RVEF, age at CMR, donor age, donor sex, ischaemic time, perfusion time, and logarithmic time since transplantation.

## Discussion

This blinded follow-up study (Trial NCT04311346) generated baseline CMR data for rejection-free patients and those with acute rejection, revealing the following key findings: (i) global T1 and T2 values consistently declined over the 24-month post-transplant period; (ii) T1 values increased during acute rejection; and (iii) dd-cfDNA levels positively associated with T1 and T2 values.

CMR's high diagnostic accuracy offers a non-invasive approach for the early detection of allograft rejection, which is essential for improving graft survival after heart transplantation. To further strengthen CMR’s role in rejection diagnostics, establishing normative data is essential. To our knowledge, this is the first prospective follow-up study to concurrently assess three outcome markers—clinical findings, EMB, and dd-cfDNA—while comparing them with CMR parameters in a cohort of heart transplant patients, both with and without rejection. To establish CMR data specifically for rejection-free heart transplant recipients, we excluded studies reporting acute rejection (ACR or AMR ≥ 1) in EMB or clinically, as well as those with elevated dd-cfDNA (≥0.15%), decreased LVEF (<40%), or evidence of rejection within the past month.

We observed a gradual recovery from transplant-related myocardial injury, evidenced by a decline in T1 and T2 values over the course of 2-year follow-up period. This extended follow-up confirms patterns seen in previous research, which noted similar improvements over shorter periods of 5–12 months.^10,18^ Elevated T1 and T2 values in CMR parametric mapping are known to reflect underlying myocardial changes, such as oedema and inflammation, highlighting the tissue-level recovery occurring over time. During the peri-transplant phase, the donor heart undergoes multiple insults that contribute to injury, such as brain death, myocardial ischaemia, and reperfusion. This is in line with our findings, as we detected LGE in 62% of patients who underwent post-transplant contrast imaging, suggesting significant myocardial injury. The prevalence of LGE in our study was at the higher end of the previously reported range (62% vs. 18–69%), whereas the extent of LGE was lower (6.5% vs. 12–18%).^19–21^ This discrepancy is likely attributable to our inclusion of minimal LGE involvement (1–4%) in the analysis.

Consequently, it is not surprising that heart transplant patients exhibit higher T1 and T2 values compared to healthy controls, even in the absence of rejection signs.^5,6,10,18^ Notably, our observation of higher T2 values in children compared to adults represents a novel contribution to the existing literature. This difference may result from variations in peri-transplant injury or age-related biochemical and structural changes. Although previous studies have yielded conflicting results regarding the impact of age on these values in healthy volunteers, none have specifically compared T1 and T2 values between children and adults.^22–25^

During acute rejection, we observed higher T1 values compared to rejection-free patients. This finding aligns with previous studies, which have demonstrated that both past and present rejection, as well as decreased LVEF, are associated with elevated T1 and T2 values^3–8,10,11^ Furthermore, we found that the difference in T1 values was evident across all regions of the left ventricle. However, during acute rejection, elevated T2 values were observed exclusively in children. This may be due to non-adherence to immunosuppressive medication in most affected children, with two showing ACR3R in EMB. Such complex rejection was not observed in adults. This suggests that while T1 mapping accurately detects acute rejection, elevated T2 values may indicate more severe rejection.

Studies involving healthy volunteers have demonstrated higher T1 values in the apical, septal, and inferior segments.^23,24^ Similarly our study found elevated T1 values in the septal and inferior regions; however we observed lower apical T1 values compared to basal segments, a pattern that aligns with one previous research.^26^ Furthermore, we observed higher T2 values in both apical and septal segments. In addition to partial volume and motion effects, the regional variations and their differences in T1 and T2 mapping may be influenced by differences in myocardial perfusion. Basal and septal perfusion are higher,^27^ while the apex experiences lower perfusion and perfusion pressure, which can lead to microvascular congestion and increased interstitial fluid accumulation. Furthermore, myocardial thickness is greater at the base, and the basal region may contain more collagen due to the higher mechanical load compared to the apex.

Some CMR studies and numerous echocardiographic studies have emphasized the connection between compromised myocardial strain and both acute rejection and unfavourable long-term outcomes.^7,8,10,11,28,29^ In our study, impaired GLS was associated with higher T2 values, while T1 values showed an association only in univariable analysis. These findings underscore the importance of incorporating myocardial functional imaging into the post-transplant follow-up protocol for heart transplant recipients.

dd-cfDNA, a novel biomarker often referred to as a ‘liquid biopsy’, is emerging as a valuable tool for identifying acute rejection in transplant patients and may reduce the need for routine surveillance EMB.^12^ However, the relationship between dd-cfDNA and T1 and T2 values in heart transplant recipients has not been thoroughly investigated. Since both dd-cfDNA levels and T1 and T2 values tend to rise during episodes of acute rejection, we hypothesized that these metrics would be associated. Our study confirmed this, showing that patients with elevated T1 and T2 values also had higher levels of dd-cfDNA.

### Limitations

While our study provides valuable insights, it is important to acknowledge its limitations. The sample size and number of rejections were relatively small, and there was no healthy control group. Additionally, the median number of CMR scans per patient was only four, and limited longitudinal data prevented us from including rejection treatment in the analysis. We were also missing dd-cfDNA samples at the 24-month time point. Nonetheless, this is one of the largest CMR report in heart transplant patients. Furthermore, we included a rejection-free control group, and EMB was performed within a median of one day after the CMR. Use of different MRI vendors for children and adults introduces bias, a known challenge in clinical settings. We addressed this with phantom-derived correction factors, which accounted for T1 and T2 relaxation biases and aligned closely with corrections from 11 healthy controls. The extension of the protocol to additional sites should include derivation of similar correction factors for the relaxation values. Despite this, other error sources may affect relaxation thresholds. The single-centre nature of the study limits the generalizability of our findings to other centres. Nonetheless, it is important to note that our tertiary medical centre conducts all heart transplants for both adults and children in Finland, which enhances the relevance of our findings.

To further validate CMR as a screening tool for rejection, prospective multicentre studies with serial CMR scans and multi-parametric rejection criteria should assess its ability to detect rejection early and guide timely interventions.

## Conclusion

This national cohort study presents several novel insights into the dynamics of T1 and T2 values in CMR for heart transplant patients. As indicators of myocardial recovery, global T1 and T2 values showed a significant decrease over a 24-month period, with notably higher values found during acute rejection episodes. The integration of CMR analysis and dd-cfDNA could provide a robust non-invasive strategy for the early detection of acute rejection.

## Supplementary Material

jeaf145_Supplementary_Data

## Data Availability

The data underlying this article cannot be shared publicly due to ethical reasons. The data are available for review by onsite visitors.
