# Supplementary material for: Cardiac magnetic resonance in heart transplant recipients: histological, clinical and cell-free DNA validation
Source: Eur Heart J Cardiovasc Imaging. 2025 May 19;26(8):1420–8. doi: 10.1093/ehjci/jeaf145 (PMC12311155; doi:10.1093/ehjci/jeaf145)
Supplement: jeaf145_Supplementary_Data [file jeaf145_supplementary_data.docx]

**Supplementary data**

| **Section** | **Title** | **Page** |
| --- | --- | --- |
| **Supplementary methods** | **CMR acquisition: cine and LGE imaging** | **2** |
| **Supplementary methods** | **CMR image analyses** | **3** |
| **Supplementary methods** | **EMB and histology** | **3** |
| **Supplementary methods** | **dd-cfDNA** | **4** |
| **Figure S1** | **T1 and T2 image analysis** | **5** |
| **Figure S2** | **LGE image analysis** | **5** |
| **Figure S3** | **Regional T1 and T2 values in children without rejection** | **6** |
| **Table S1** | **Univariable and multivariable analysis of variables influencing T1 mapping values** | **7** |
| **Table S2** | **Univariable and multivariable analysis of variables influencing T2 mapping values** | **8** |

**Supplementary methods**

**CMR acquisition: cine and LGE imaging**

The measurement protocol included short-axis and long-axis 2D balanced steady-state free precession (bSSFP) cine imaging. The short-axis images covered the whole heart with stack typically consisting of 17 slices. The long-axis images were acquired in 2- and 3-chamber directions with one slice and in 4-chamber, and right ventricle outflow tract (RVOT) directions with three slices. The slice thickness was 6 mm in all cine images, with a typical acquisition pixel size of 1.7 x 1.7 mm with pediatric patients and 1.5 x 1.5 mm with adults. If patient was not able to maintain sufficient breath hold, additional short-axis scan was made in free breathing with three signal averages to suppress motion artefacts. For the cine images, retrospective ECG triggering was used as a primary method, with prospective triggering used as an alternative method to suppress image artefacts due to arrhythmia if necessary.

Late gadolinium enhancement (LGE) imaging with Dotarem (279.3 mg/ml, 0.4 ml/kg, max 40 ml) was done by using phase-sensitive inversion recovery (PSIR) sequence with bSSFP readout in short-axis, long-axis, and LVOT directions. Whole heart covering short-axis views were typically consisting of 10 slices and long-axis views were acquired in 2- and 4-chamber directions. The slice thickness in all directions were 8 mm with a typical acquisition pixel size of 2.0 x 2.0 mm with pediatric patients and 1.9 x 1.9 mm with adults. The correct inversion time was verified by using TI-scout sequence before the first LGE sequence. The first LGE imaging was performed as early as possible after transplantation and repeated at 1- and 2-year post-transplant.

**CMR image analyses**

CMR analyses were performed using Medis Suite 4.0 (Medis Medical Imaging Systems, Leiden, the Netherlands) to assess motion corrected T1 times, T2 times, volumetric data, and strain parameters. Right and left ventricle ejection fractions, as well as measurements of systolic left ventricular global longitudinal (GLS), and global circumferential (GSC) strain were obtained with semiautomated calculations. The ventricular contours were manually adjusted, when necessary. A senior radiologist (L.M.) and the senior cardiologist, who primarily interpreted the images, reanalyzed 10 randomly selected CMR studies for T1- and T2-maps to assess intraobserver and interobserver variability. Both observers selected the T1 and T2 mapping series they judged to be of the highest quality, choosing either the systolic or diastolic phase for analysis. The second reviewer was blinded to both the EMB and dd-cfDNA results, as well as to the analyses conducted by the first reviewer.

**EMB and histology**

At least three endomyocardial biopsies were obtained from the right interventricular septum via a jugular, or femoral venous approach with the aid of both fluoroscopy and transthoracic echocardiography. An experienced pathologist, blinded to the CMR and dd-cfDNA results interpreted samples according to the International Society of Heart and Lung Transplantation (ISHLT) 2005 and 2013 criteria, and categorized them into ACR grade 0R, 1R, 2R, or 3R, and AMR grade pAMR0, pAMR1 (H+), pAMR1 (I+), pAMR2, or pAMR3. For the study analysis, AMR was reclassified into two groups: no AMR (pAMR0) and AMR (≥ pAMR1). Immunohistochemistry was performed on all EMBs according to institutional guidelines, systematically in the early post-transplant period and at later time points if AMR was suspected clinically or histologically. Routine hematoxylin-eosin stained slides from formalin-fixed and paraffin embedded tissue blocks were prepared from the samples. For immunohistochemistry, the slides were deparaffinized, and heat-induced epitope retrieval, counterstaining, dehydrating, clearing, and mounting were performed following standard protocol. The antibody binding site was visualized using a DAB and/or RED detection kit (Roche, Basel, Switzerland). The C4d (clone SP91) antibody was from Abcam (Cambridge, UK), and antibodies for CD31 (clone JC70A) and CD68 (clone PG-M1) were from Dako (Santa Clara, USA). The immunostainers used were Agilent DAKO Autostainer Link 48 (Santa Clara, USA) and Roche Ventana The BenchMark ULTRA (Roche, Basel, Switzerland).

**dd-cfDNA**

Venous blood for ddPCR-based dd-cfDNA analysis were collected at scheduled clinical follow-ups and upon signs of rejection. Levels of the circulating cfDNA was assessed by QX200 Droplet Digital PCR System (Bio-Rad, USA) with up to 3 heterologous SNPs per patient. Assays were performed as instructed by the manufacturer. The resulting data were analyzed by the QuantaSoft Analysis Pro software (version 1.0, Bio-Rad, USA). The dd-cfDNA percentages were calculated by dividing the number of droplets positive for the donor allele by the total number of droplets positive for both the donor and reference alleles. When the donor genotype was available, the droplet count of a heterozygous donor allele was multiplied by 1.33 and the droplet count of its reference allele was adjusted by subtracting the corrected donor count from the reference droplet count.

**Figure S1
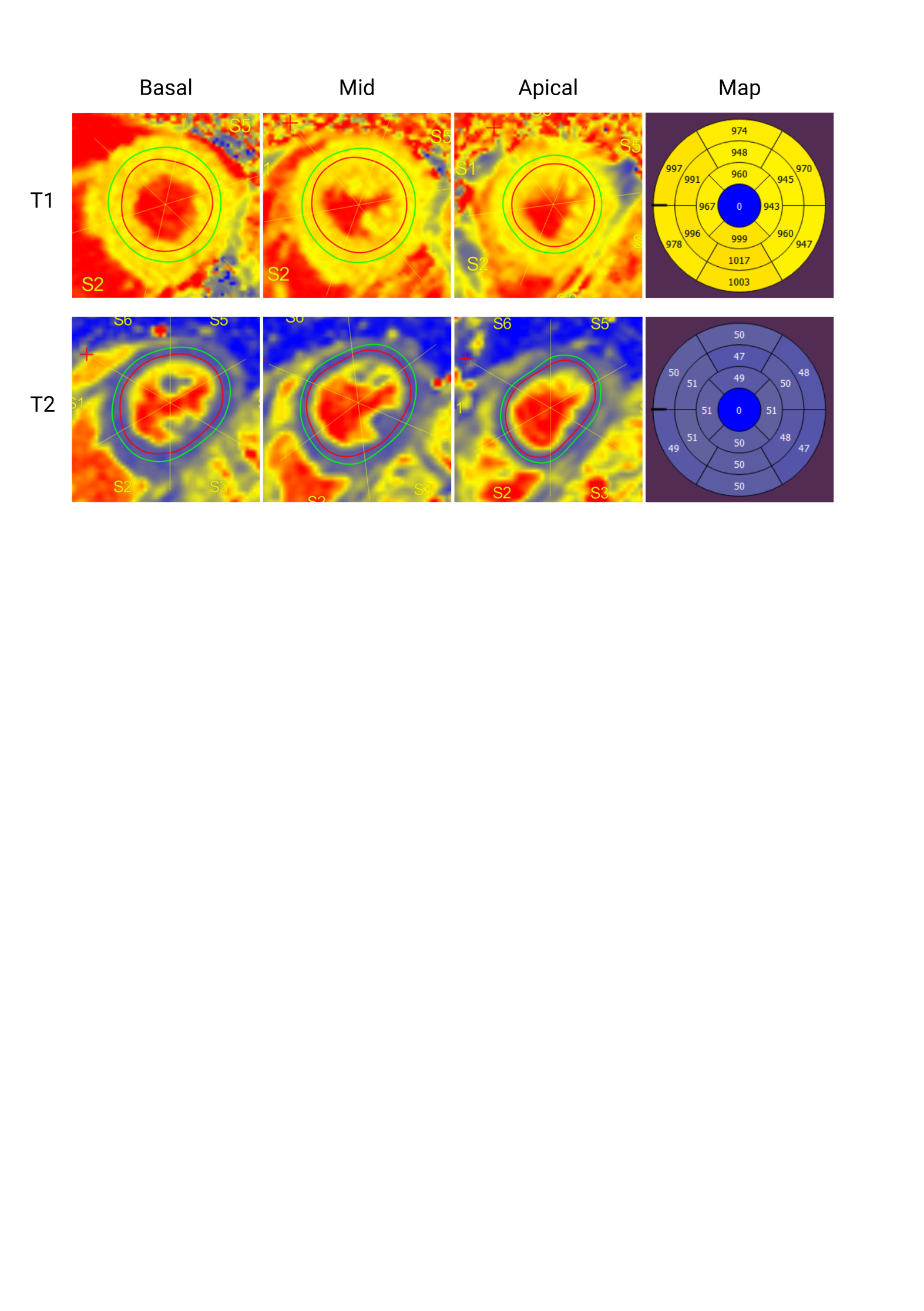
**

**T1 and T2 image analysis: from ROI tracing to relaxation time maps.**

**Figure S2**

**
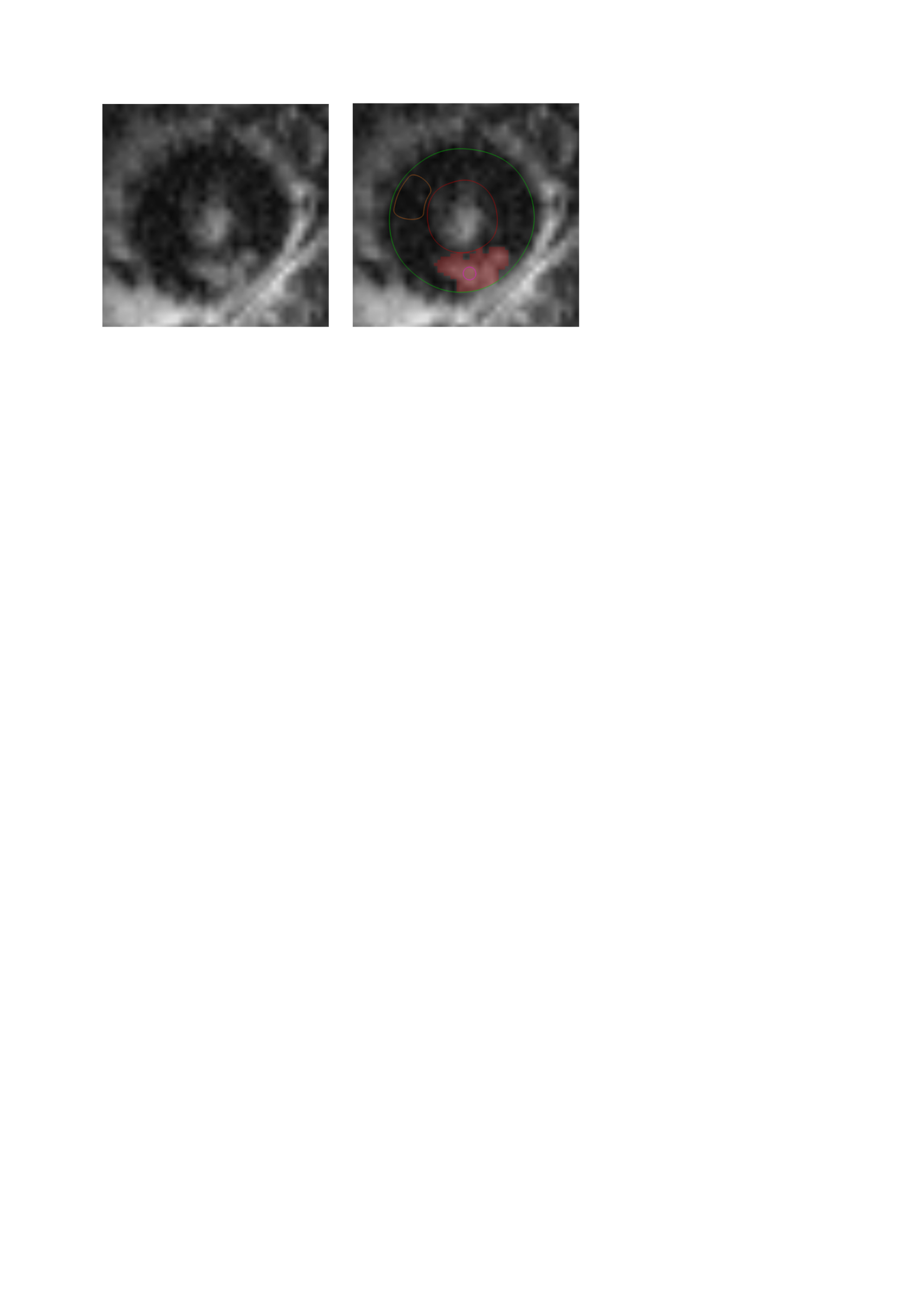
**

**LGE image analysis: full width at half maximum method, with manual adjustments as required.**

**Figure S3**

**
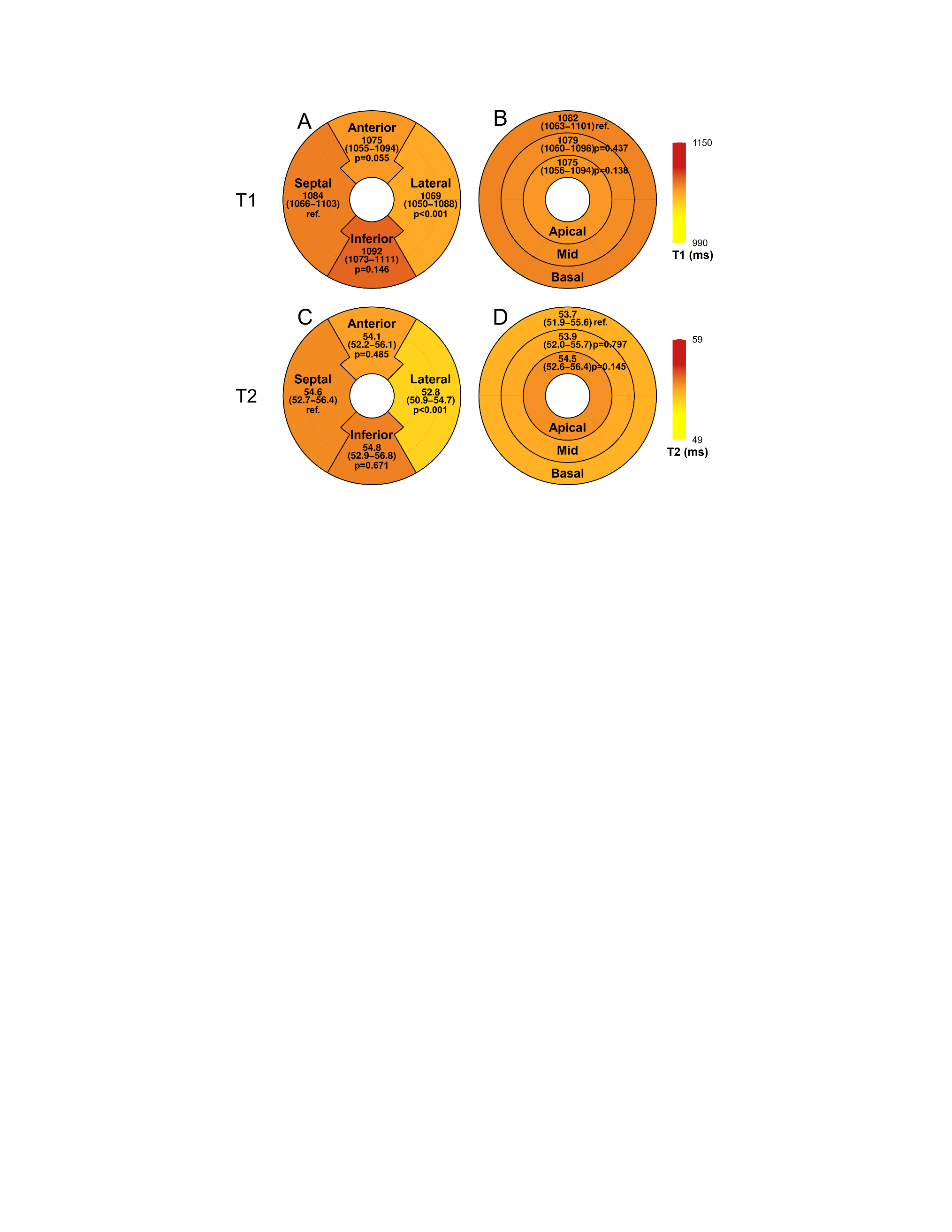
**

**Regional T1 and T2 values in children without rejection**

Regional T1 mapping (A and B) and T2 mapping (C and D) linear mixed-effects model estimates (95% CI) and comparisons in rejection-free children at one month post-transplant. (A and C) Septal segments compared to anterior, lateral, and inferior segments. (B and D) Basal segments compared to midventricular and apical segments.

**Table S1**

**Univariable and Multivariable Analysis of Variables Influencing T1 Mapping Values**

|  | **Univariable T1** | | **Multivariable T1** | |
| --- | --- | --- | --- | --- |
|  | **Beta (95% CI)** | **P value** | **Beta (95% CI)** | **P value** |
| Age at CMR | -0.52 (-0.92 to -0.11) | **0.013** | -0.53 (-1.35 to 0.29) | 0.200 |
| Time since HTx, log(mo) | -8.92 (-10.37 to -7.46) | **<0.001** | -3.12 (-5.46 to -0.79) | **0.009** |
| dd-cfDNA per 0.1 | 5.88 (4.53 to 7.23) | **<0.001** | 5.24 (3.79 to 6.68) | **<0.001** |
| GLS | 0.64 (0.32 to 0.97) | **<0.001** | 0.15 (-0.27 to 0.58) | 0.475 |
| GCS | 0.59 (0.32 to 0.86) | **<0.001** | 0.42 (-0.01 to 0.84) | 0.056 |
| LVEF | -0.39 (-0.69 to -0.09) | **0.012** | 0.25 (-0.34 to 0.84) | 0.400 |
| RVEF | -0.51 (-0.81 to -0.21) | **<0.001** | -0.60 (-1.03 to -0.16) | **0.007** |
| Donor age | 0.23 (-0.44 to 0.91) | 0.489 | 1.16 (-0.03 to 2.35) | 0.056 |
| Donor sex, male | -5.40 (-25.53 to 14.73) | 0.593 | -14.76 (-40.31 to 10.80) | 0.249 |
| Ischemic time, min | 0.01 (-0.14 to 0.17) | 0.855 | 0.13 (-0.06 to 0.31) | 0.169 |
| Perfusion time, min | 0.09 (-0.02 to 0.20) | 0.114 | 0.12 (-0.06 to 0.31) | 0.180 |

Linear mixed-effects model results using vendor-corrected values for T1. Multivariable analysis includes adjustment for all variables shown in the univariable model. The bold values denote statistically significant differences.

CMR, cardiac magnetic resonance imaging; dd-cfDNA, donor-derived cell-free DNA; GCS, global circumferential strain; GLS, global longitudinal strain; HTx, heart transplantation; LVEF, left ventricular ejection fraction; RVEF, right ventricular ejection fraction

**Table S2**

**Univariable and Multivariable Analysis of Variables Influencing T2 Mapping Values**

|  | **Univariable T2** | | **Multivariable T2** | |
| --- | --- | --- | --- | --- |
|  | **Beta (95% CI)** | **P value** | **Beta (95% CI)** | **P value** |
| Age at CMR | -0.07 (-0.10 to -0.04) | **<0.001** | -0.04 (-0.09 to 0.01) | 0.104 |
| Time since HTx, log(mo) | -0.44 (-0.55 to -0.32) | **<0.001** | 0.17 (-0.02 to 0.36) | 0.077 |
| dd-cfDNA per 0.1 | 0.10 (-0.01 to 0.21) | 0.065 | 0.14 (0.03 to 0.25) | **0.014** |
| GLS | 0.05 (0.03 to 0.08) | **<0.001** | 0.10 (0.07 to 0.14) | **<0.001** |
| GCS | 0.01 (-0.01 to 0.03) | 0.506 | -0.00 (-0.04 to 0.03) | 0.892 |
| LVEF | 0.00 (-0.02 to 0.03) | 0.680 | -0.00 (-0.05 to 0.04) | 0.926 |
| RVEF | 0.02 (-0.01 to 0.04) | 0.162 | 0.02 (-0.02 to 0.05) | 0.292 |
| Donor age | -0.03 (-0.09 to 0.02) | 0.196 | 0.07 (0.00 to 0.14) | **0.046** |
| Donor sex, male | -2.00 (-3.49 to -0.50) | **0.010** | -2.14 (-3.65 to -0.62) | **0.007** |
| Ischemic time, min | -0.01 (-0.02 to 0.01) | 0.360 | -0.01 (-0.02 to 0.00) | 0.239 |
| Perfusion time, min | 0.01 (0.00 to 0.02) | **0.004** | 0.01 (0.00 to 0.02) | **0.013** |

Linear mixed-effects model results using vendor-corrected values for T2. See Table S2 for footnote.
